# Supplementary material for: Restoration of fertility in nonablated recipient mice after spermatogonial stem cell transplantation
Source: Stem Cell Reports. 2024 Mar 7;19(4):443–55. doi: 10.1016/j.stemcr.2024.02.003 (PMC11096438; doi:10.1016/j.stemcr.2024.02.003)
Supplement: Document S1. Figure S1, Tables S1, S2, and supplemental experimental procedures [file mmc1.pdf]

Stem Cell Reports, Volume *19*

Supplemental Information

Restoration of fertility in nonablated recipient mice after spermatogonial stem cell transplantation

Hiroko Morimoto, Narumi Ogonuki, Shogo Matoba, Mito Kanatsu-Shinohara, Atsuo Ogura, and Takashi Shinohara

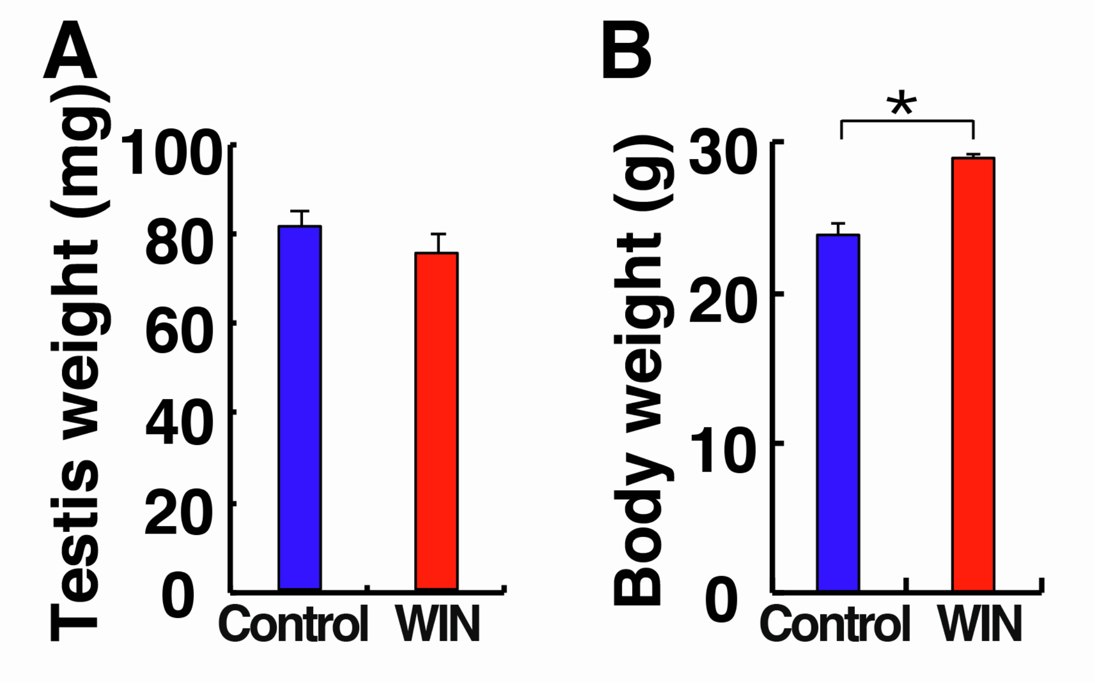

Supplemental Figure S1 The impact of WIN on testis and body size. (A) Testis weight (n = 6), (B) Body weight (n = 3).

Supplemental Table S1 Antibodies used in the present study

name	company	catalog number	application
Mouse anti ACTB monoclonal antibody	Sigma Aldrich: St. Lois, MO	A5441	Western blot (1.4 µg /ml)
Rabbit anti mouse CLDN3 polyclonal antibody	Thermo Fisher: Rockford, IL	34-1700	Immunostaining Western blot (0.5 µg /ml)
Rabbit anti human CLDN5 monoclonal antibody	abcam : Cambridge, UK	ab131259	Western blot (2.6 µg /ml)
Rabbit human CLDN5 polyclonal antibody	abcam : Cambridge, UK	ab15106	Immunostaining (1/200)
Rabbit anti human CLDN11 polyclonal antibody	abcam : Cambridge, UK	ab53041	Immunostaining Western blot (1.0 µg /ml)
Rabbit anti human CXCL12 polyclonal antibody	SantaCruz : Dallas, TX	sc-28876	Immunostaining Western blot (2.0 µg /ml)
Goat anti human FGF2 polyclonal antibody	SantaCruz : Dallas, TX	sc-1390	Immunostaining (2.0 µg /ml)
Mouse anti human FGF2 monoclonal antibody	SantaCruz : Dallas, TX	sc-74412	Western blot (0.5 µg /ml)
Rabbit anti human GDNF polyclonal antibody	SantaCruz : Dallas, TX	sc-328	Immunostaining (0.25 µg /ml)
Rabbit anti mouse GDNF polyclonal antibody	abcam : Cambridge, UK	ab18956	Western blot (5.0 µg /ml)
Rabbit anti-mouse OCLN	Gift from Dr. Sachiko Tsukita (Osaka Univ)	Not applicable	Immunostaining (1.0 µg /ml))

Mouse anti human OCLN monoclonal antibody	SantaCruz : Dallas, TX	sc-133256	Western blot (0.5 µg /ml)
Rabbit anti mouse SYCP3	NOVUS, Centennial, CO,	NB300-231	Immunostaining (10.0 µg /ml)
APC streptavidin	Biolegend : San Diego, CA	#405207	Biotin Tracer (0.2 µg /ml)
Alexa Fluor 488 donkey anti rat IgG	Thermo Fisher: Rockford, IL	A21208	Immunostaining (2.0 µg /ml)
Alexa Fluor 488 donkey anti goat IgG	Thermo Fisher: Rockford, IL	A11055	Immunostaining (2.0 µg /ml)
Alexa Fluor 555 donkey anti mouse IgG	Thermo Fisher: Rockford, IL	A31571	Immunostaining (2.0 µg /ml)
Alexa Fluor 555 donkey anti rabbit IgG	Thermo Fisher: Rockford, IL	A31573	Immunostaining (2.0 µg /ml)
HRP-anti rabbit IgG	Cell signaling: Danvers, MA	#7074	Western blot (1/2000)
HRP-anti muse IgG	Cell signaling: Danvers, MA	#7076	Western blot (1/2000)
Rhodamine conjugated Peanut agglutinin	Vector laboratories: Burlingame, CA	#RL-1072-5	Immunostaining (1/500)

Supplemental Table S2 PCR primers used in the present study

primer	forward	reverse
Cldn3	CACCACTACCAGCAGTCGATGAAC	AGACTGTGTGTCGTCTGTCACCATC
Cldn5	TAACCTGAAAGGGCAGCTGGAGAAAC	AGGGTCCAGGCTAAGTCCTTTGGTTCAGTAG
Cldn11	CTGCCGAAAAATGGACGAACTG	TGCACGTAGCCTGGAAGGATGA
Cxcl12	GCTCTGCATCAGTGACG	CCAGGTACTCTTGGATCC
Fgf2	CTCTACTGCAAGAACGGCG	CATAGCAAGGTACCGGTTGG
Gdnf	GCCACTTGGAGTTAATGTCC	CTTCGAGAAGCCTCTTACCG
Hprt	GCTGGTGAAAAGGACCTCT	CACAGGACTAGAACACCTGC
Ocln	TTGGGACAGAGGCTATGG	ACCCACTCTTCAACATTGGG

Supplemental experimental procedure

Analysis of recipient testes

Recipient mice were sacrificed 2 months after transplantation, and the number of donor cell-derived colonies was counted under UV light. Clusters of germ cells were considered as colonies when they exceeded a length of 0.1 mm and extended across the entire basement membrane of the seminiferous tubules, as previously defined (Nagano et al., 1999). For evaluation of regeneration, two histological sections were prepared from the testes and epididymides of each animal. The number of tubule cross-sections showing spermatogenesis (defined as the presence of multiple layers of germ cells in entire circumference of the seminiferous tubules) was recorded by counting all the tubules on the section. For epididymal tubule count, tubules containing any sperm were recorded. Perimeter was determined by cellSens (Olympus, Tokyo, Japan).

Tracer experiment

Biotin tracer experiments were conducted following established procedures (Takashima et al., 2011). In brief, sulfo-NHS-LC-biotin solution (7.5 mg/ml; Thermo Fisher Scientific, Waltham, MA) was prepared in phosphate-buffered saline (PBS) containing 1 mM $CaCl_2$. Approximately 10 μl of the biotin solution was microinjected into the interstitium of adult (> 8-week-old) testes using a syringe with 30 G disposable needles. After 30 min, the testes were collected and fixed in 4% paraformaldehyde. The samples were frozen in liquid nitrogen for cryosectioning. Frozen sections were prepared

for further staining by allophycocyanin (APC)-conjugated streptavidin (Biolegend, San Diego, CA). Endogenous biotin staining was examined by incubation with APC-conjugated streptavidin without biotin injection.

Immunostaining

Testis samples were fixed in 4% paraformaldehyde for 2 h at 4°C, and embedded in Tissue-Tek OCT compound (Sakura Finetek, Tokyo, Japan) for cryosectioning. Sections of 8 μm thickness were made. To block non-specific antibodies, sections were treated with 3% bovine serum albumin (BSA) and 10% goat serum in PBS supplemented with 0.1% Tween 20 (PBST) for 1 h at room temperature. The sections were then incubated with indicated primary and secondary antibodies with 0.5 % BSA in PBST, overnight and for 1 h, respectively. Thirty tubules were counted from at least two mice. Rhodamine-labeled PNA (Vector Laboratories, Burlingame, CA) was used to detect the acrosome. Antibodies used are listed in Table S1. Hoechst 33342 (Sigma) was used for counterstaining. Staining without the primary antibodies were used as negative controls.

Real-time PCR analysis

Total RNA was isolated using TRIzol reagent (Invitrogen, Carlsbad, CA). First-strand cDNA was produced using a Verso cDNA synthesis kit for reverse transcription-PCR (Thermo Fisher Scientific). For real-time PCR, CFX connect™ real-time PCR system (Bio-Rad, Hercules, CA) and *Power* SYBR Green PCR Master Mix (Applied Biosystems) were utilized according to the manufacturers' protocols. Transcript levels

were normalized to those of *Hprt*. The PCR conditions were as follows: 95°C for 10 min, followed by 40 cycles at 95°C for 15 s and 60°C for 1 min. Each PCR was performed at least in triplicate. Primers used are listed in Table S2.

Western blotting

Samples were separated by SDS-PAGE, transferred to Immobilon-P membranes (Millipore, Burlington, MA), and incubated with primary antibodies. The antibodies used in the experiments are shown in the Table S1.

Microinsemination

EGFP-expressing seminiferous tubules were collected, and the germ cells were mechanically extracted from the tubules using fine forceps. Microinsemination was performed as described previously using elongated spermatids and sperm (Ogonuki et al., 2006). Embryos that reached the two-cell stage after 24 h in culture were transferred to the oviducts of day-1 pseudopregnant ICR female mice. Fetuses were retrieved on day 19.5 by caesarean section.

Supplemental references

Nagano, M., Avarbock, M. R., and Brinster, R. L. (1999). Pattern and kinetics of mouse donor spermatogonial stem cell colonization in recipient testes. Biol. Reprod. *60*, 1429-1436.

Ogonuki, N., Mochida, K., Miki, H., Inoue, K., Fray, M., Iwaki, T., Moriwaki, K., Obata, Y., Morozumi, K., Yanagimachi, R., and Ogura, A. (2006). Spermatozoa and spermatids retrived from frozen reproductive organs or frozen whole bodies of male mice can produce offspring. Proc. Natl. Acad. Sci. USA *103*, 13098-13103.

Takashima, S., Kanatsu-Shinohara, M., Tanaka, T., Takehashi, M., Morimoto, H., and Shinohara, T. (2011). Rac mediates mouse spermatogonial stem cell homing to germline niches by regulating transmigration through the blood-testis barrier. Cell Stem Cell *9*, 463-475.
