# Supplementary material for: Restoration of fertility in nonablated recipient mice after spermatogonial stem cell transplantation
Source: Stem Cell Reports. 2024 Mar 7;19(4):443–55. doi: 10.1016/j.stemcr.2024.02.003 (PMC11096438; doi:10.1016/j.stemcr.2024.02.003)
Supplement: Document S2. Article plus supplemental information [file mmc2.pdf]

Stem Cell Reports
Article
OPEN ACCESS

Restoration of fertility in nonablated recipient mice after spermatogonial stem cell transplantation

Hiroko Morimoto,[1] Narumi Ogonuki,[2] Shogo Matoba,[2] Mito Kanatsu-Shinohara,[1,3] Atsuo Ogura,[2] and Takashi Shinohara[1,4,*]
[1]Department of Molecular Genetics, Graduate School of Medicine, Kyoto University, Kyoto 606-8501, Japan
[2]Bioresource Engineering Division, RIKEN BioResource Research Center, Ibaraki 305-0074, Japan
[3]AMED-CREST, AMED 1-7-1 Otemachi, Chiyodaku, Tokyo 100-0004, Japan
[4]Lead contact
*Correspondence: tshinoha@virus.kyoto-u.ac.jp
https://doi.org/10.1016/j.stemcr.2024.02.003

SUMMARY

Spermatogonial stem cell (SSC) transplantation is a valuable tool for studying stem cell-niche interaction. However, the conventional approach requires the removal of endogenous SSCs, causing damage to the niche. Here we introduce WIN18,446, an ALDH1A2 inhibitor, to enhance SSC colonization in nonablated recipients. Pre-transplantation treatment with WIN18,446 induced abnormal claudin protein expression, which comprises the blood-testis barrier and impedes SSC colonization. Consequently, WIN18,446 increased colonization efficiency by 4.6-fold compared with untreated host. WIN18,446-treated testes remained small despite the cessation of WIN18,446, suggesting its irreversible effect. Offspring were born by microinsemination using donor-derived sperm. While WIN18,446 was lethal to busulfan-treated mice, cyclophosphamide- or radiation-treated animals survived after WIN18,446 treatment. Although WIN18,446 is not applicable to humans due to toxicity, similar ALDH1A2 inhibitors may be useful for SSC transplantation into nonablated testes, shedding light on the role of retinoid metabolism on SSC-niche interactions and advancing SSC research in animal models and humans.

INTRODUCTION

The study of stem cell-niche interaction is a well-established field, with numerous investigations spanning several decades (Tikhonova et al., 2020). The niche is known to provide factors essential for the self-renewal division of stem cells in an undifferentiated state (Schofield, 1978). However, the lack of stem cell-specific markers and the scarcity of stem cells present a significant challenge to understanding the mechanism underlying niche control of stem cells. Transplantation assays have emerged as a valuable tool for studying this problem in the study of hematopoietic stem cells (HSCs). By introducing HSCs into an animal, researchers can assess donor cell colonization. Although studying the hematopoietic environment is challenging due to the limited accessibility of the bone marrow, the fundamental concept of stem cell-niche interactions has emerged from these transplantation studies and is now extensively utilized in the context of other self-renewing tissues.

Spermatogenesis represents another self-renewing tissue where similar functional transplantation assays can be applied. Spermatogonial stem cells (SSCs) continuously divide, forming foundation of spermatogenesis (de Rooij and Russell, 2000; Meistrich and van Beek, 1993). Positioned on the basement membrane of the seminiferous tubules, SSCs generate committed progenitor cells continuously. Transplanting SSCs into vacant seminiferous tubules leads to the colonization and regeneration of donor SSCs (Brinster and Zimmermann, 1994). Recipient animals are typically prepared using busulfan, a chemical agent that eradicates SSCs in the host's testes. After being introduced into the adluminal cavity of the busulfan-treated mouse testes, transplanted SSCs migrate between the blood-testis barrier (BTB), which consists of Sertoli cells. Once they settle on the basement membrane, spermatogenesis reinitiates, eventually culminating in sperm production. In the most successful cases, offspring are produced through natural mating (Brinster and Avarbock, 1994).

Comparing the morphological estimates of SSC numbers with the results from functional transplantation assay has indicated that only a restricted number of SSCs can effectively reconstitute the recipient microenvironment. It is estimated that approximately 5%–10% of transplanted SSCs colonize the seminiferous tubules (Nagano et al., 1999; Ogawa et al., 2003). Despite the low efficiency of transplantation in empty seminiferous tubules, SSC colonization can occur in nonablated mice (Morimoto et al., 2021). However, the number of colonies originating from donor SSCs was notably restricted, with around 20% of the colonies compared with those in busulfan-treated empty seminiferous tubules. Moreover, while germ cell colonies in busulfan-treated testes gradually develop mature sperm, colonies in nonablated mice are generally smaller and limited in differentiation levels, possibly due to the influence of endogenous spermatogenesis. Consequently, while stem cell-niche interactions may exhibit relative flexibility in the testis, they still do not offer sufficient support

This is an open access article under the CC BY license (http://creativecommons.org/licenses/by/4.0/).

for efficient donor colonization to produce an adequate amount of sperm for offspring production.

The decreased colonization efficiency is believed to be partly attributed to the BTB. Normally, spermatogenic cells move from the basal compartment of the seminiferous tubules to the adluminal compartment. However, transplanted SSCs migrate in the opposite direction, from the adluminal compartment to the basal compartment. Notably, the BTB, which is composed of several tight junction proteins (TJPs), including OCLN, CLDN3, CLDN5, and CLDN11, is critical for meiosis and protection against autoimmunity (Wu et al., 2020). The involvement of the BTB in spermatogonial transplantation was initially proposed in experiments using immature recipients. Transplantation was performed before the BTB formation that occurs around 2 weeks after birth. These studies reported an approximately 5-fold increase in donor cell colonization (Shinohara et al., 2001). Furthermore, another study directly confirmed the involvement of CLDN11 in SSC colonization. The absence of the BTB in *Cldn11* knockout (KO) mice enhanced colonization efficiency by approximately 3.3-fold (Kanatsu-Shinohara et al., 2020). Building upon these insights, we attempted to enhance colonization using small interfering RNA (siRNA) (Morimoto et al., 2021). In this experiment, siRNA was administered before cell transplantation into the seminiferous tubules followed by transplantation 4 days later. While this treatment did improve colony formation, it only resulted in a few colonies in a single testis. Due to the necessity for multiple microinjections and the low colonization efficiency, manipulating TJP manipulation with siRNA proved impractical.

An alternative strategy to manipulate the BTB is through specialized chemicals. Given that permanent disruption of the BTB interferes with spermatogenesis, transient BTB suppression is a preferred approach. Several studies have reported that inhibiting retinoic acid (RA) signaling disrupts the BTB (Hasegawa and Saga, 2012; Chihara et al., 2013; Kent et al., 2016; Jauregui et al., 2018). RA is crucial for meiosis, and a deficiency in RA inhibits the differentiation of undifferentiated spermatogonia into KIT^+ differentiating spermatogonia (Gewiss et al., 2021). RA also plays a vital role in the BTB. Transfecting a dominant negative RA receptor gene into Sertoli cells resulted in BTB disruption during stages VII–XII of spermatogenesis (Hasegawa and Saga, 2012). These results raised a possibility that the administration of RA inhibitor may transiently disrupt the BTB. One of the useful tools was WIN18,446 (WIN), an inhibitor of ALDH1A2, a testis-specific isoform of the enzyme involved in RA metabolism, which transiently inhibited spermatogenesis (Amory et al., 2011). Importantly, sperm counts and fertility recovered after treatment were discontinued. The utility of WIN in spermatogonial transplantation was reported in a more recent study, in which busulfan-treated mice sired offspring via WIN administration (Nakamura et al., 2021). The authors proposed that WIN suppressed differentiation of donor cells and enhanced colonization. Unfortunately, however, the possibility of the BTB disruption was not examined in this study (Nakamura et al., 2021). Moreover, WIN is toxic to busulfan-treated mice, leading to the death of ∼40% of recipient mice during the experiments (Morimoto et al., 2023).

In the current study, we aimed to assess the potential of WIN in enhancing donor cell colonization in nonablated, wild-type recipients. Given that WIN exhibited limited toxicity in untreated wild-type mice, we hypothesized that a transient disruption of the BTB in nonablated wild-type mice enhance colonization without significantly compromising the health of the recipient animals. Additionally, because RA deprivation is known to reduce SSC numbers (McLean et al., 2002), we anticipated that WIN treatment alone could create vacant niches for donor SSCs. This study involved administering WIN to nonablated recipient mice to explore the possibility of using these animals as recipients for spermatogonial transplantation.

RESULTS

WIN administration inhibits spermatogonial differentiation

To assess the impact of WIN on the normal testicular environment, we followed a previously established protocol and subcutaneously injected 12-week-old mice with WIN for 13 days (Nakamura et al., 2021). At this stage, the testes that received WIN injections did not show significant changes in testis weight (Figure S1A), although it increased body weight (Figure S1B). However, when comparing the ratio of testis weight to body weight, WIN-treated testes showed a significantly smaller weight (Figure 1A). Histological examination revealed a partial inhibition of spermatogenesis in WIN-treated mice (Figure 1B). The perimeter of the seminiferous tubules was significantly reduced (Figure 1C).

To identify the types of remaining cells, we conducted immunostaining (Figure 1D). GFRA1 is expressed in A_{single} (A_s), A_{paired} (A_{pr}), and some $A_{aligned}$ (A_{ali}) undifferentiated spermatogonia (Grisanti et al., 2009). CDH1 is expressed in the entire population of undifferentiated spermatogonia (Tokuda et al., 2007), while KIT is expressed in differentiating spermatogonia (Yoshinaga et al., 1991). Quantitative analysis of stained cells revealed a significant reduction in the number of KIT^+ differentiating spermatogonia (Figure 1D). In contrast, there was an increase in the number of $CDH1^+$ undifferentiated spermatogonia and a decrease in $GFRA1^+$ undifferentiated spermatogonia. Despite these

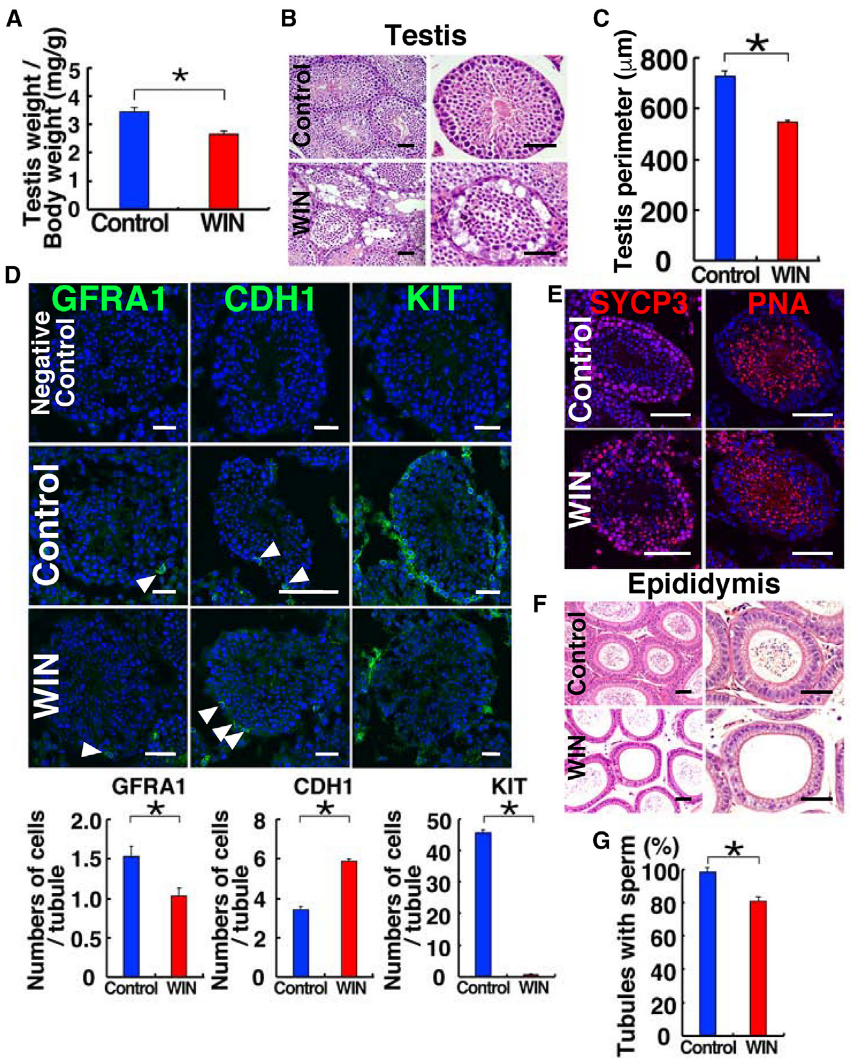

Figure 1. Suppression of spermatogenesis in wild-type mice after consecutive administration of WIN for 13 days

(A) Testis/body weight ratio (n = 6 from three mice).

(B) Histological appearance of testes after WIN administration.

(C) Perimeter of the seminiferous tubules (n = 30 from three mice).

(D) Immunostaining of wild-type testes using antibodies against spermatogonia markers. Quantification of spermatogonia numbers (n = 30 from two mice).

(E) Immunostaining of meiotic (SYCP3) and haploid (PNA) cell markers.

(F and G) Histological appearance of epididymides after WIN administration and the number of tubules with sperm (n = 20 for WIN; n = 24 for control from two mice). Bar, 50 μm (B and F), 30 μm (D and E). Stain: hematoxylin and eosin (B and F), Hoechst33342 (D and E). See also Figure S1 and Table S1.

changes in the spermatogonia compartment, the WIN-treated testes still contained SYCP3$^+$ spermatocytes and peanut agglutinin (PNA)$^+$ haploid cells (Figure 1E). However, histological analysis of the epididymis showed that the number of epididymal tubules with sperm was significantly reduced (Figures 1F and 1G). Since GFRA1 is considered more specific to SSCs than CDH1, these findings suggest that 13 days of WIN treatment increases the number of committed undifferentiated spermatogonia while decreasing the SSC population.

Increased FGF2 expression and altered CXCL12 localization by WIN administration

To assess the influence of WIN on the SSC microenvironment, we examined the expression of *Gdnf*, *Fgf2*, and *Cxcl12*. *Gdnf* and *Fgf2* are self-renewal factors for SSCs (Kanatsu-Shinohara and Shinohara, 2013), while *Cxcl12* is a chemokine involved in homing of SSCs into a germline niche (Kanatsu-Shinohara et al., 2012). Real-time PCR analysis revealed a slight increase in *Fgf2* mRNA due to WIN treatment (Figure 2A). A more substantial increase was observed in *Cxcl12* mRNA. However, western blot analysis showed a significant rise in FGF2 expression following WIN treatment (Figure 2B).

Because it was possible that the changes in cytokine expression pattens were caused by the different composition of WIN-treated testes, we then assessed the distribution of these cytokines through immunostaining (Figure 2C). FGF2 expression patterns remained relatively stable, with FGF2 signals appearing stronger in WIN-treated mice. They were distributed along the basement membrane of the seminiferous tubules. GDNF signals, which were located along the basement membrane and in interstitial tissues, did not exhibit apparent changes.

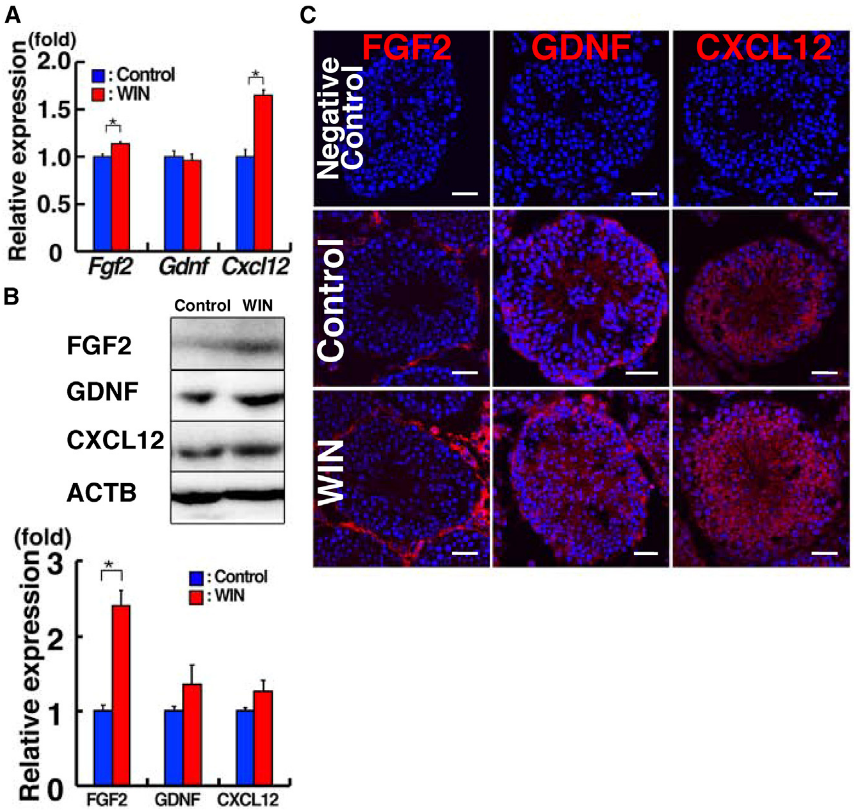

Figure 2. Elevated FGF2 expression following WIN treatment
(A) Real-time PCR analysis of cytokines (n = 3 from three mice).
(B) Western blot analysis of WIN-treated testis (n = 3 from three mice).
(C) Immunostaining of WIN-treated testes. Bar, 30 μm (C). Stain: Hoechst33342 (C). See also Tables S1 and S2.

However, CXCL12 signals appeared most pronounced in the adluminal compartment in WIN-treated mice. While we also observed stronger signals in the adluminal compartments in control mice, these signals were found in both the basal and adluminal compartments in WIN-treated mice. Although these changes might have occurred as a result of the changes in the ratio of the cell types in WIN-treated testes, they raised a possibility that WIN might influence self-renewal and homing to their niches.

Functional analysis of WIN on SSCs

Given that WIN causes irreversible damage to spermatogenesis and vitamin A deprivation reduces SSC numbers (McLean et al., 2002; Paik et al., 2014), the decrease in GFRA1^{+} spermatogonia numbers suggested that WIN might induce apoptosis in SSCs (Figure 3A). To test this possibility, we conducted terminal deoxynucleotidyl transferase dUTP nick end labeling (TUNEL) staining (Figure 3B). The TUNEL staining showed an increase in apoptosis in WIN-treated animals. However, immunostaining with a GFRA1 antibody showed that these cells did not express GFRA1.

Although these findings suggested that WIN does not induces apoptosis in SSCs, conflicting reports regarding GFRA1 expression in SSCs exist (Ebata et al., 2005; Buageaw et al., 2005). In addition, apoptosis of GFRA1^{+} cells might have escaped detection analysis because analysis was performed only at one time point. Therefore, we utilized spermatogonial transplantation, a reliable functional assay for SSCs (Brinster and Zimmermann, 1994). Testis cells were collected from 8-week-old C57BL6/Tg14(act-EGFP-OsbY01) (designated green) mice that ubiquitously express *Egfp* transgene. These mice received a 13-day WIN treatment. Unlike the initial experiments (Figure 1A), the size of the testes treated with WIN was notably smaller (Figure 3C). The more dramatic effects found in green mice may reflect the fact that they received the same dose of WIN (2 mg per mouse) at a younger age (8 weeks). As anticipated due to the reduced testis size, the number of recovered testis cells significantly decreased with WIN treatment (Figure 3D). These cells were microinjected into the seminiferous tubules of busulfan-treated testes.

Analysis of the testis revealed a significant increase in the number of colonies resulting from the transplantation of WIN-treated mice (Figure 3E and 3F), indicating that WIN treatment increases the concentration of SSCs in the donor testis cells (Figure 3F). However, when the total SSC number was calculated by multiplying total cell recovery by the SSC concentration (i.e., colony number), WIN-treated animals and control animals showed comparable numbers of SSCs (800.1 vs. 921.3 per testis) (Figure 3G), which was

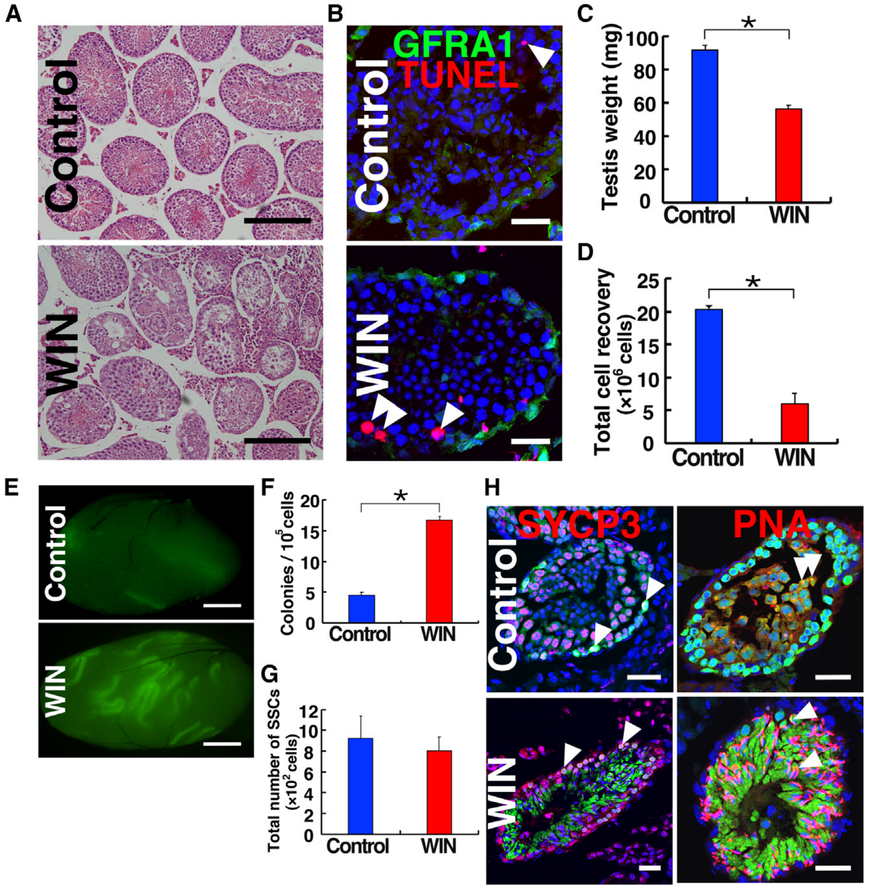

Figure 3. Functional analysis of SSCs in WIN-treated testes

(A) Histological appearance of donor testis used for transplantation.
(B) TUNEL staining of WIN-treated testes.
(C) Testis weight (n = 4 from three mice).
(D) Total cell recovery (n = 3 from three mice).
(E) Macroscopic appearance of recipient testis transplanted with WIN-treated donor cells.
(F) Colony count (n = 17 for WIN from nine mice; n = 18 for control from nine mice). Results from six experiments.
(G) Total SSC number (n = 17 for WIN from nine mice; n = 18 for control from nine mice). Results from six experiments.
(H) Immunostaining of recipient testes showing cells expressing SYCP3 (red) and PNA (red). Bar, 200 μm (A), 30 μm (B and H), 1 mm (E). Stain: hematoxylin and eosin (A), Hoechst33342 (B and H). See also Table S1.

within the range of previously reported results (Oatley et al., 2011; Kanatsu-Shinohara et al., 2016; Boyer et al., 2021). Immunostaining displayed normal spermatogenesis with SYCP3$^+$ spermatocytes and PNA$^+$ haploid cells (Figure 3H). Assuming that a loss of specific germ cell types did not occur during the cell isolation and transplantation, these results indicate that WIN does not influence SSC numbers and maintains normal differentiation potential after spermatogonial transplantation. Therefore, WIN likely does not induce irreversible damages to germ cells.

Analysis of TJP expression in WIN-treated mice

We then examined the feasibility of using WIN-treated mice as recipients. We analyzed the effects of WIN on TJPs because TJPs comprise the BTB and influence SSC colonization (Kanatsu-Shinohara et al., 2020). In addition, previous studies suggested that disturbance in RA signaling impairs the BTB (Hasegawa and Saga, 2012), which was confirmed in our previous study (Morimoto et al., 2023). Therefore, WIN may also influence SSC homing in untreated testes. On the other hand, it was also possible that full spermatogenesis in wild-type mice may not allow such colonization. We first analyzed the expression by real-time PCR (Figure 4A). Although we did not find significant changes in *Cldn3*, *Cldn5*, and *Cldn11* expression, *Ocln* expression was significantly downregulated by WIN treatment. To confirm these mRNA results, we next carried out western blot analysis. Surprisingly, the expression of CLDN11 and OCLN increased significantly in WIN-treated testes (Figure 4B).

We proceeded with immunostaining of WIN-treated testes (Figure 4C). Consistent with previous reports, not only Sertoli cells but also germ cells expressed several types of claudin proteins (Morrow et al., 2009; Takashima et al., 2011). There was a significant downregulation of CLDN5 around the BTB. Conversely, we noted significant increases in CLDN11 and OCLN expression. Overall, the location of their signals did not seem to change significantly, but the staining intensity was stronger around the basal compartment where the BTB is formed between Sertoli cells. These results suggest that WIN disrupts the function of the BTB.

To directly assess this possibility, we evaluated the permeability of the BTB by injecting biotin into the interstitial tissue (Figure 4D). In contrast to the control mice, numerous seminiferous tubules in WIN-treated mice contained biotin.

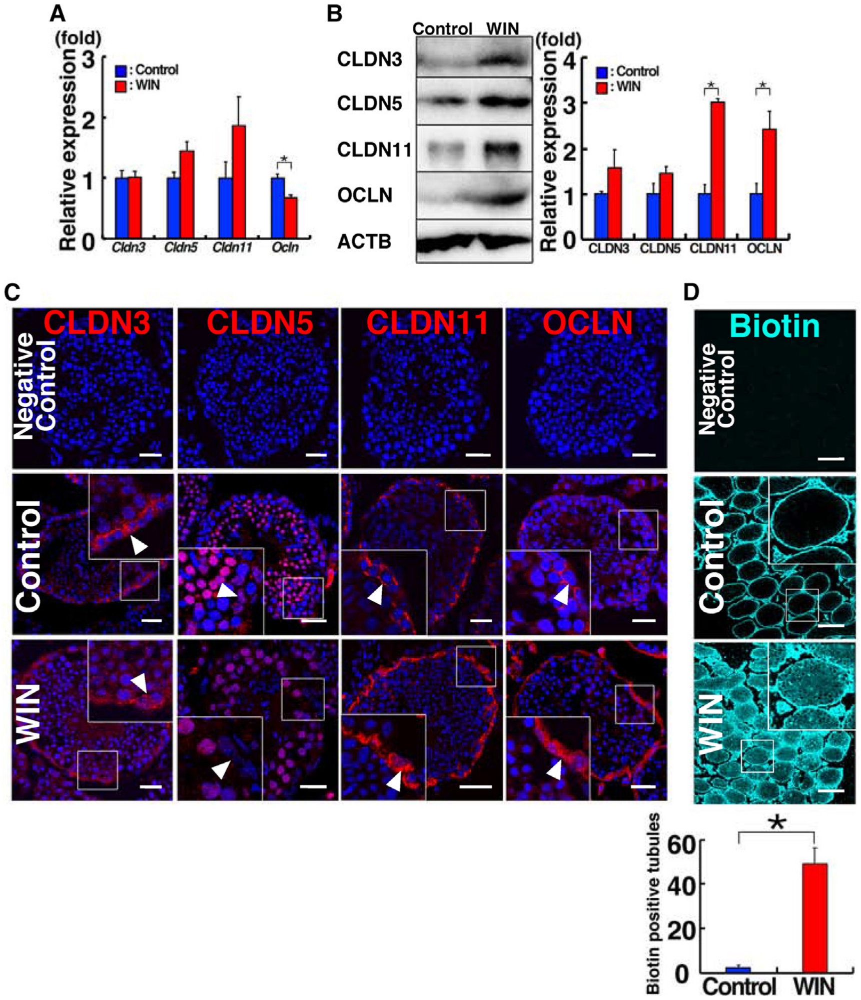

Figure 4. Impaired BTB following WIN treatment

(A and B) Real-time PCR (A; n = 4 mice for WIN; n = 3 mice for control) and western blot (B; n = 3 mice) analyses of TJPs.

(C) Immunostaining of WIN-treated testes.

(D) Functional assessment of the BTB (n = 423 for WIN; n = 375 for control from three mice). WIN-treated testes underwent interstitial injection of biotin. Thirty minutes after microinjection, the testes were fixed and sectioned. Biotin was detected by APC-conjugated streptavidin (cyan). Bar, 30 μm (C), 200 μm (D). Stain: Hoechst33342 (C and D). See also Tables S1 and S2.

These findings imply that WIN may enhance SSC colonization by modulating the BTB function in wild-type testes.

Functional analysis of SSCs in WIN-treated testes by serial transplantation

To test this possibility, we collected testes from green mice and transplanted the cells 3 days after starting WIN treatment. The recipient mice received additional WIN for 10 days. Two months after transplantation, the number of colonies were counted (Figure 5A). The morphology of the colony in both WIN-treated and control testes was significantly different from those in busulfan-treated testes. The former consisted of a heterogeneous distribution of EGFP$^+$ cells with diverse morphology. In contrast, EGFP$^+$ cells distributed relatively homogeneously in the busulfan-treated secondary recipient testes (Figure 5B). In addition, we also observed a lasting impact of WIN on testis weight, suggesting an irreversible effect on the testis. Even though the recipient mice were assessed 3 months after transplantation (i.e., approximately 10 weeks after WIN treatment), the testes that had been exposed to WIN were still smaller than the control testes (Figure 5C), despite undergoing two cycles of spermatogenesis (35 days). Quantification of colonies revealed a significant increase in colony number after WIN treatment (Figure 5D). These results showed that WIN treatment of wild-type recipient mice enhances colonization but induces an irreversible effect on the testis.

To determine the degree of self-renewal division in transplanted SSCs, we performed serial transplantation. We dissociated the recipient testis cells (primary recipients) into single cells. These cells were then transplanted into busulfan-treated secondary recipient mice, which were not treated with WIN. When these mice were killed 2 months after transplantation, the number of colonies was determined, and the degree of self-renewal division was assessed. Since each colony is derived from single SSC and approximately 10% of transplanted SSCs colonize busulfan-treated testes (Nagano et al., 1999; Kanatsu-Shinohara et al., 2006),

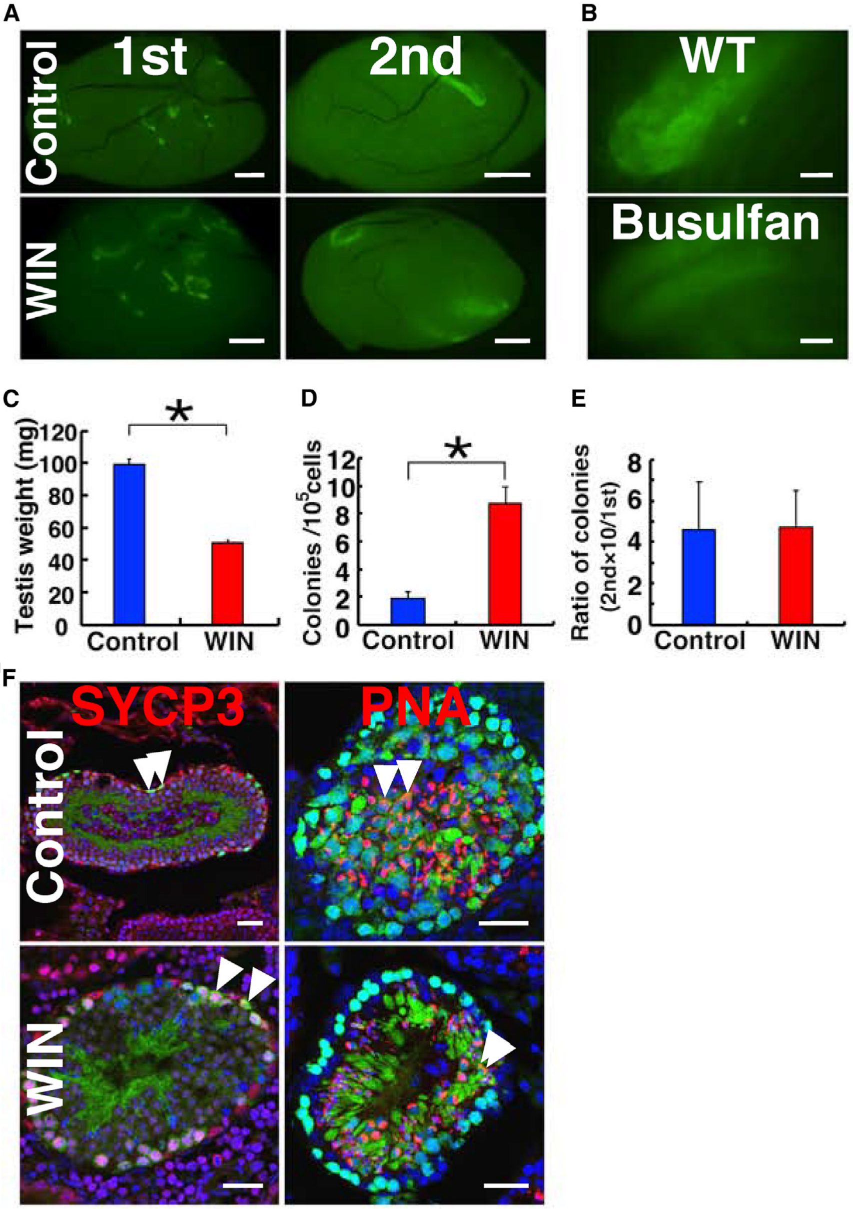

Figure 5. Serial transplantation of SSCs in WIN-treated testes

(A) Macroscopic appearance of recipient testes. Note the difference in colony morphology between primary recipients (nonablated) and secondary recipients (busulfan-treated).

(B) Appearance of colonies in wild-type or busulfan-treated mice. Colonies in busulfan-treated testes are homogeneous and exhibit symmetrical cell distribution, while those in nonablated testes display heterogeneous cell distribution patterns with variable degrees of vertical differentiation.

(C) Testis weight of the primary recipient mice (n = 4 for WIN; n = 5 for control from four mice).

(D) Colony count in the primary recipients (n = 23 for WIN from 12 mice; n = 29 for control from 15 mice). Results from seven experiments.

(E) Total increase in colony numbers (total regenerated colony number × 10)/(primary colony number used for serial transplantation) (n = 18 for WIN; n = 9 for control). Results from 18 to nine experiments, respectively, for WIN and control mice.

(F) Immunostaining of the secondary recipients showing cells expressing SYCP3 (red) and PNA (red). Bar, 1 mm (A), 125 μm (B), 30 μm (F). Stain: Hoechst33342 (E). See also Table S1.

the ratio of secondary colonies per primary colonies was 4.7 ± 1.8 and 4.6 ± 2.3 for WIN-treated and control testis cells, respectively (Figure 5E). The difference was not statistically different. Recipient testes showed normal spermatogenesis with SYCP3$^+$ spermatocytes and PNA$^+$ haploid cells (Figure 5F). These results demonstrate that WIN treatment does not affect the self-renewal division in the primary recipients.

Birth of normal offspring by microinsemination

To determine whether sperm generated in WIN-treated mice are fertile, we conducted microinsemination 4 months after transplantation (Figure 6A). Although some colonies remained small and undifferentiated, others appeared relatively long and contained multiple layers of germ cells, suggesting the presence of haploid cells. We selected these elongated colonies with differentiating germ cells. We collected sperm and elongated spermatozoa from the cell suspensions and microinjected these cells into the oocytes to produce offspring.

A total of 123 embryos were created using sperm generated from three recipient testes (Figure 6B). Out of these, 55 embryos developed to the 2-cell stage after 24 h of culture. Thirty-nine embryos were transferred into the

A

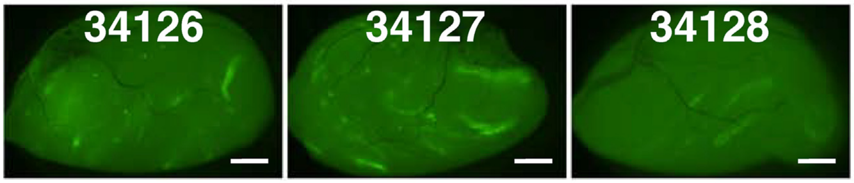

B

sample	No. of constructed embryo	No. of cleaved/ cultured(%)	No. of embryos transferred	No. of implanted (% transferred)	No. of offspring (% transferred)	EGFP
34126	19	6 (31.5)	6	2 (33.3)	1 (16.7)	0
34127	53	27 (50.9)	11	6 (54.5)	1 (9.1)	1
34128	51	22 (43.1)	22	13 (59.1)	4 (18.2)	2

C

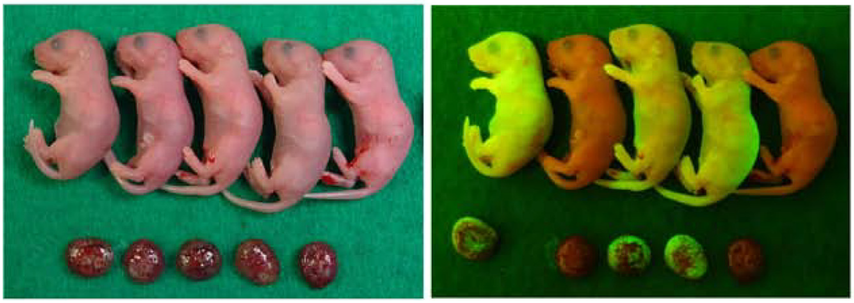

Figure 6. Restoration of fertility in non-ablated recipients through microinsemination

(A) Macroscopic appearance of the WIN-treated recipient mouse testes used for microinsemination.

(B) *In vitro* development of reconstituted embryos.

(C) Appearance of offspring at the time of birth. Bar, 1 mm (A).

oviducts of pseudopregnant mothers. After cesarean delivery, 21 embryos implanted into the uteri, resulting in the birth of six offspring. Although one of the offspring was found dead at the time of the cesarean delivery, three out of the five live offspring displayed EGFP fluorescence when exposed to UV light (Figure 6C). These results demonstrate that sperm generated in WIN-treated mice exhibit normal functionality.

Effect of WIN administration after chemical and radiation treatment

WIN may be beneficial for the treatment of infertility in cancer patients who have lost sperm due to cancer treatments. However, our previous analysis showed that WIN kills ∼45% of busulfan-treated mice, limiting its applicability. Therefore, we assessed the impact of other cytotoxic treatments on the health of WIN-treated mice. We selected cyclophosphamide and radiation, both of which affect spermatogenesis and are widely used in cancer treatment.

Thirty-five days after administration of each chemical, we treated the animals with WIN for 13 days. This corresponds to one cycle of spermatogenesis (35 days), and we examined the effect of WIN after the depletion of germ cells during regeneration. A total of 10 and nine mice were used to evaluate the toxicity of cyclophosphamide and irradiation, respectively. We administered cyclophosphamide at 200 mg/kg. This dose of cyclophosphamide eliminates differentiating spermatogonia and the early preleptotene stage but retains SSCs (Drumond et al., 2011). In contrast to busulfan treatment, none of the cyclophosphamide-treated mice died upon WIN administration (Figure 7A). On the other hand, irradiation of mice with 6 Gy induces a partial loss of endogenous SSCs and allows donor cell colonization (Ishii et al., 2014). Although one mouse died, the rest of the mice survived despite continuous WIN treatment (Figure 7A).

Thirteen days after WIN treatment, testes of WIN-treated mice were significantly smaller in both groups (Figure 7B). Histological analysis of the testes showed numerous tubules with germ cells in cyclophosphamide-treated mice regardless of WIN treatment (Figure 7C). Although spermatogenesis was somewhat disorganized, all the tubules contained germ cells. However, radiation treatment had a more significant impact. In control mice, although some tubules were totally empty, the remaining tubules exhibited normal spermatogenesis. In contrast, WIN-treated testes contained germ cells with variable degrees of differentiation. Although spermatozoa were found in the epididymis of cyclophosphamide-treated mice, no spermatozoa were found after irradiation. Thus, unlike busulfan, cyclophosphamide or irradiation does not appear to exert severe toxicity when combined with WIN treatment.

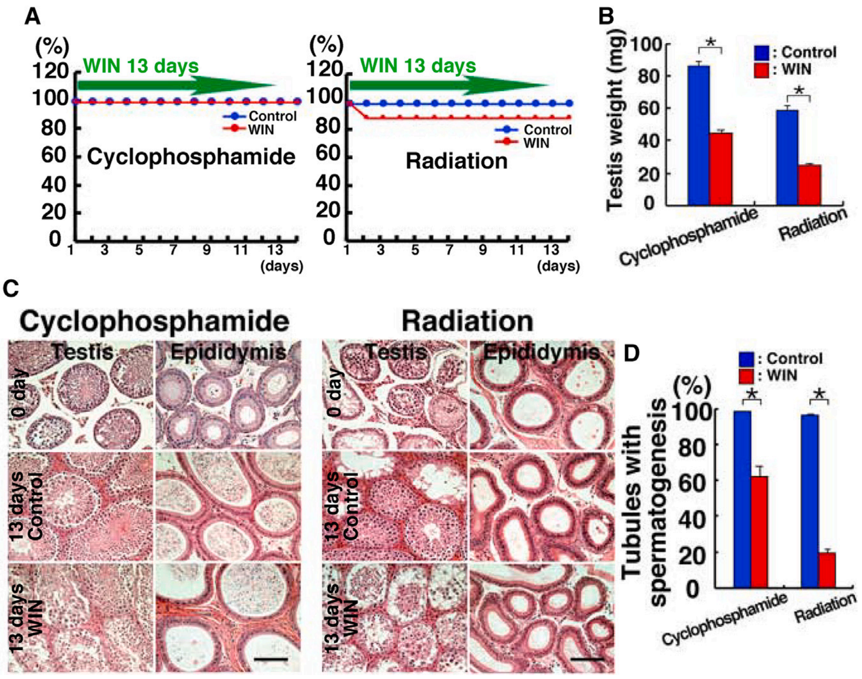

Figure 7. Mortality of WIN-treated mice after irradiation or chemical treatment

(A) Survival of cyclophosphamide-treated (left; n = 10) or irradiated (right; n = 9) mice. Thirty-five days after treatment with cyclophosphamide or irradiation, mice were administered WIN for 13 days.

(B) Testis weight after cyclophosphamide (n = 20) or irradiation (n = 16 for WIN; n = 18 for control) treatment.

(C) Histological appearance of testes treated with cyclophosphamide (left) or radiation (right) after WIN administration.

(D) The number of tubules with spermatogenesis after cyclophosphamide (n = 1,219 for WIN; n = 1,845 for control) or irradiation treatment (n = 950 for WIN; n = 1,359 for control from two mice). Bar, 100 μm (C). Stain: hematoxylin and eosin (C).

DISCUSSION

The BTB posed a significant obstacle for SSCs when they were microinjected into the adluminal compartment (Shinohara et al., 2001; Kanatsu-Shinohara et al., 2020). Previously, WIN was shown to be effective in increasing donor cell colonization in busulfan-treated mice by suppressing differentiation during spermatogonial transplantation (Nakamura et al., 2021); however, it remained unclear whether WIN could enhance colonization in wild-type testes, where a significant number of endogenous SSCs already occupy their niches. If these endogenous cells were similarly suppressed in their differentiation, donor cells would not be able to find empty niches. Of greater concern was the fact that WIN had been associated with a mortality rate of ~45% among injected animals after busulfan treatment (Morimoto et al., 2023). This study was initiated to assess the feasibility of using WIN to enhance SSC colonization in wild-type mice without inducing chemical castration.

RA is essential for spermatogonia differentiation and meiosis (Gewiss et al., 2021). However, a previous study showed that RA depletion decreases the number of SSCs (McLean et al., 2002). Therefore, we hypothesized that WIN treatment might create empty niches in wild-type seminiferous tubules filled with mature germ cells. However, another study using immature mice showed that WIN expands the number of SSCs (Agrimson et al., 2017). In these experiments, WIN increased the number of ID4^{bright} cells, which are enriched for SSCs. In contrast, WIN decreased the number of ID4^{dim} cells, which are probably progenitor cells. Therefore, the impact of WIN on immature and mature testes may not be the same depending on the age of the animals. Because WIN treatment reduced the number of GFRA1^{+} undifferentiated spermatogonia in adult testes, we initially thought that WIN might destroy endogenous SSCs. However, our transplantation experiments did not show significant changes in SSC number when we treated green mice with WIN and analyzed the number of SSCs. These results suggested that 13 days of WIN treatment is insufficient for creating empty niches for donor SSCs.

Our findings in this study confirm our previous observations on the disparity between mRNA and protein levels (Morimoto et al., 2021, 2023). For instance, we noted that busulfan treatment upregulated *Gdnf* mRNA while leaving *Fgf2* mRNA unchanged (Morimoto et al., 2021); however, western blot analysis showed downregulation of GDNF and FGF2. In the current study, we showed that WIN upregulated *Fgf2* mRNA and FGF2 protein in untreated wild-type testes, which is consistent with the antagonism between RA and FGF in many tissues (del Corral and Storey, 2004). No changes were found for *Gdnf* mRNA and GDNF protein. Therefore, GDNF and FGF2 are regulated by germ cells and RA metabolism in distinct ways. Our cytokine expression analysis suggests complex regulation of self-renewal factor regulation. Unlike busulfan-treated testes, WIN does not completely eliminate endogenous

germ cells. As GDNF did not change significantly in WIN-treated mice and most of the differentiating spermatogonia stage are removed after WIN treatment, we speculate that undifferentiated spermatogonia stimulates GDNF expression. This possibility needs to be investigated in future experiments.

We also studied the impact of WIN on the BTB. This analysis revealed distinct expression levels of claudin mRNA and protein, which may be due to posttranscriptional regulation or rapid turnover (Holczbauer et al., 2013). Notably, the expression patterns of several TJPs, which comprise the BTB, changed dramatically due to WIN treatment. However, the pattern of the change was not exactly the same as the busulfan-treated mice. In busulfan-treated mice, WIN disrupted the BTB by changing the expression patterns of CLDN3, CLND5, CLDN11, and OCLN (Morimoto et al., 2023). While normal Sertoli cells expressed these proteins, a significant proportion of Sertoli cells changed expression of TJPs after WIN treatment. However, not all Sertoli cells were influenced by WIN. It is possible that WIN administration for longer periods of time might have eliminated claudin expression completely. Alternatively, because RA signaling is periodically activated at stages VII–XII, it is possible that WIN may not influence all TJP signals. The striking difference in the staining patterns of TJPs suggested that germ cells have a significant impact on TJP expression. Despite the difference in TJP expression patterns, the permeability of the BTB was compromised regardless of germ cells. Although the increased permeability of the BTB appears counterintuitive, some of the claudin proteins, such as CLDN2, are known as pore-forming claudins (Günzel and Alan, 2013). Moreover, inter-claudin interactions in both *cis* (in the same cell) and *trans* (in adjacent cells) are required for proper claudin polymerization. For example, CLDN4 overexpression disrupts higher order CLDN2 structures, reduces CLDN2 anchoring at the tight junction (Shashikanth et al., 2022). Therefore, increased expression of specific claudins might have contributed to increase the permeability of the BTB. These results suggested that not only the RA metabolism but also germ cells have a significant role in the regulation of the BTB.

Transplantation into WIN-treated testes clearly demonstrated an enhancement of donor cell colonization. We observed more than a 4-fold increase in colony numbers compared with control DMSO-treated hosts (8.7 vs. 1.9 per 10^6 cells). The level of colonization was comparable to that found in *Cldn11* KO mouse recipients without busulfan treatment (9.1 per 10^6 cells) (Kanatsu-Shinohara et al., 2020). Considering that WIN can only transiently disrupt the BTB, the extent of enhancement is remarkable. In a typical spermatogonial transplantation experiment, approximately 10^6 cells can be microinjected into the busulfan-treated testis, resulting in the production of ∼20 colonies (Nagano et al., 1999). This represents the maximum level of colonization without donor cell enrichment. Therefore, our results suggested that around 40% to 50% of SSCs can colonize the seminiferous tubules without the aid of busulfan. While enhanced donor stem cell colonization can be achieved in an untreated hematopoietic system, it is generally necessary to repeat transplantation to enhance donor HSC colonization (Bretcher et al., 1982). In this sense, our result suggests more flexible interaction between stem cells and niches in the testis. It will be interesting to examine whether the efficiency can be improved by transplantation of cultured SSCs, which can be derived from a small testis piece (Kanatsu-Shinohara et al., 2003).

Our serial transplantation experiments showed no impact on self-renewal due to WIN treatment. Although we observed an increased number of colonies in the primary recipients, the ratio of secondary colonies to primary colonies did not change significantly. This suggests that the number of SSCs did not significantly increase in the primary recipients. This result aligns with the transplantation of WIN-treated donor cells, where the total number of SSCs per testis did not change significantly despite the significant reduction in total cell recovery by WIN treatment. It is not surprising that a large number of endogenous SSCs did not provide sufficient empty niches and limited their expansion. Considering that RA deficiency decreases SSC number (McLean et al., 2002), it is possible that longer WIN treatment before SSC transplantation may lead to more efficient colonization and self-renewal division.

Although the efficiency of colonization in WIN-treated mice is still relatively low, it was sufficient to apply the microinsemination technique (Ogura et al., 2005). However, in the case of nonablated recipients, microinsemination is technically demanding because not all germ cell colonies were fully differentiated. In untreated wild-type testes, only a few colonies are typically found, making it challenging to find fully matured colonies (Morimoto et al., 2021). However, the increased colonization facilitated by WIN allowed us to collect a sufficient number of sperm, resulting in the successful production of offspring from unablated hosts. Although the birth of offspring demonstrated the fertility of donor-derived sperm, it should be noted that they may not be completely normal because microinsemination can cause transgenerational abnormalities in mice (Kanatsu-Shinohara et al., 2023).

While our results suggest the potential utility of WIN in spermatogonial transplantation into nonablated wild-type testes, the toxicity of WIN makes it impossible to use this chemical for clinical applications. Because the mechanism for this toxicity remains unclear, pathological analysis of the dead mice is important for understanding the

mechanism of toxicity. However, it is still possible that a similar chemical might also exhibit the same type of toxicity. We were curious to know whether the toxicity of WIN is specific to busulfan. Our preliminary experiments with cyclophosphamide or radiation-treated mice did not result in mortality. Therefore, the toxicity of WIN may be influenced by the type and dose of the drugs. In addition, the route of administration may also be critical. For example, oral administration suppressed weight gain, while we observed increased body weight after subcutaneous injection (Haenisch et al., 2021). Because WIN is not toxic to untreated animals, busulfan probably affects the expression of other enzymes that are involved in oxidative stress (DeLeve and Wang, 2000; Myers et al., 2017); however, another concern for WIN is the irreversible effect on testis size. Although complete spermatogenesis was found, the testis size remained small despite cessation of WIN treatment, which is consistent with findings from a previous study (Paik et al., 2014). This report showed irreversible binding of WIN to ALDH1A2. Although we currently do not know whether such testes become normal after a longer period, we feel that the damage may be permanent because even vitamin A deprivation can cause irreversible damage after long-term treatment (Chihara et al., 2013). Because donor cells could differentiate normally in the secondary recipients, the WIN-induced damages must have occurred in somatic cells. Given that WIN can act on ALDH1A1 and ALDH1A3 (Paik et al., 2014; Arnold et al., 2015), the development of more specific RA metabolism inhibitors without irreversible effects is required for clinical application.

Spermatogonial transplantation holds promise for restoring fertility in boys who undergo cancer treatment (Kubota and Brinster, 2018). Since these patients typically lack SSCs and sperm, there will be no need for suppressing RA to empty SSC niches. However, because of the transient disruption of the BTB, suppression of the RA signaling may still enhance SSC colonization. Offspring production from nonablated recipients represents a significant advancement in SSC research, as it eliminates the need for depleting endogenous germ cells. The removal of germ cells often harms the recipient environment, as demonstrated in rats, where.the elimination of endogenous germ cells led to extensive edema (Ogawa et al., 1999). If this WIN-based spermatogonial transplantation proves effective in other animal species, it could enhance the efficiency of donor cell colonization and contribute to the refinement of spermatogonial transplantation techniques. Although WIN is toxic to use for clinical application, our findings may spark investigations into the potential use of similar RA inhibitors in SSC research, which may hopefully contribute to development of new reproductive technologies in experimental animals and humans.

EXPERIMENTAL PROCEDURES

Resource availability

Lead conatct

Further information and requests for resources and reagents should be directed to and will be fulfilled by the lead contact, Takashi Shinohara (tshinoha@virus.kyoto-u.ac.jp).

Materials availability

This study did not generate new unique reagents.

Data and code availability

This study did not generate new Datasets.

Animals and transplantation procedure

For WIN treatment, 8- to 12-week-old green mice or wild-type on a C57BL/6 (B6) background (Japan SLC, Shizuoka, Japan) received subcutaneous injections of WIN (2 mg/animal) for 13 days (2 mg; Cayman Chemical, Ann Arbor, MI), as described previously (Nakamura et al., 2021). Controls received DMSO solution. In addition to the aforementioned mice, B6 mice were used after exposure to irradiation (6 Gy), busulfan (44 mg/kg), or cyclophosphamide treatment (200 mg/kg; Wako, Kyoto, Japan). For transplantation into busulfan-treated testes, the mice were used at least 4 weeks after busulfan injection. For quantification of SSCs after WIN treatment, donor cells were prepared from green mice using a two-step enzymatic procedure with collagenase type IV and trypsin (Sigma, St. Louis, MO). In brief, seminiferous tubules were gently dissected and incubated in collagenase type IV solution for 15 min at 37°C. After rinsing the tubules twice, tubules were dissociated into single cells by incubating in trypsin (0.2%) and DNase (1.4 mg/mL) for 10 min at 37°C. Donor cells were microinjected into the seminiferous tubules of busulfan-treated mice via the efferent duct (Ogawa et al., 1997). Approximately 10^6 testis cells were injected into each testis. For serial transplantation, recipient testes were collected 2 months after the initial transplantation. After counting the colonies, the testes were dissociated into single cells using collagenase type IV and trypsin. Cells collected from a primary recipient testis were transplanted into three secondary recipient testes. Each injection filled approximately 75%–85% of the seminiferous tubules. The Institutional Animal Care and Use Committee of Kyoto University approved all animal experimentation protocols.

Statistical analyses

Significant differences between means for single comparisons were determined by Student's t test. Multiple comparisons were carried out using ANOVA followed by Tukey's honestly significant difference test.

SUPPLEMENTAL INFORMATION

Supplemental information can be found online at https://doi.org/10.1016/j.stemcr.2024.02.003.

ACKNOWLEDGMENTS

Financial support for this research was provided by MEXT (23K20043, 23H00399, 23K05781, 22K19429, 19K22512, 19H05750, 18H05281).

AUTHOR CONTRIBUTIONS

H.M., M.K.-S., and T.S. designed the experiments. H.M. carried out most of the experiments. N.O., S.M., and A.O. carried out microinsemination. T.S. performed spermatogonial transplantation. H.M., M.K.-S., and T.S. and wrote the manuscript.

DECLARATION OF INTERESTS

The authors declare no competing interests.

Received: October 30, 2023
Revised: February 2, 2024
Accepted: February 5, 2024
Published: March 7, 2024

REFERENCES

Agrimson, K.S., Oatley, M.J., Mitchell, D., Oatley, J.M., Griswold, M.D., and Hogarth, C.A. (2017). Retinoic acid deficiency leads to an increase in spermatogonial stem cell number in the neonatal testis, but excess retinoic acid results in no change. Dev. Biol. *432*, 229–236.

Amory, J.K., Muller, C.H., Shimshoni, J.A., Isoherranen, N., Paik, J., Moreb, J.S., Amory, D.W., Evanoff, R., Goldstein, A.S., and Griswold, M.D. (2011). Suppression of spermatogenesis by bisdichloroacetyldiamines is mediated by inhibition of testicular retinoic acid biosynthesis. J. Androl. *32*, 111–119.

Arnold, S.L., Kent, T., Hogarth, C.A., Schlatt, S., Prasad, B., Haenisch, M., Walsh, T., Muller, C.H., Griswold, M.D., Amory, J.K., and Isoherranen, N. (2015). Importance of ALDH1A enzymes in determining human testicular retinoic acid concentrations. J. Lipid Res. *56*, 342–357.

Boyer, A., Zhang, X., Levasseur, A., Abou Nader, N., St-Jean, G., Nagano, M.C., and Boerboom, D. (2021). Constitutive activation of CTNNB1 results in a loss of spermatogonial stem cell activity in mice. PLoS One *16*, e0251911.

Brecher, G., Ansell, J.D., Micklem, H.S., Tjio, J.H., and Cronkite, E.P. (1982). Special proliferative sites are not needed for seeding and proliferation of transfused bone marrow cells in normal syngeneic mice. Proc. Natl. Acad. Sci. USA *79*, 5085–5087.

Brinster, R.L., and Zimmermann, J.W. (1994). Spermatogenesis following male germ-cell transplantation. Proc. Natl. Acad. Sci. USA *91*, 11298–11302.

Brinster, R.L., and Avarbock, M.R. (1994). Germline transmission of donor haplotype following spermatogonial transplantation. Proc. Natl. Acad. Sci. USA *91*, 11303–11307.

Buageaw, A., Sukhwani, M., Ben-Yehudah, A., Ehmcke, J., Rawe, V.Y., Pholpramool, C., Orwig, K.E., and Schlatt, S. (2005). GDNF family receptor alpha1 phenotype of spermatogonial stem cells in immature mouse testes. Biol. Reprod. *73*, 1011–1016.

Chihara, M., Otsuka, S., Ichii, O., and Kon, Y. (2013). Vitamin A deprivation affects the progression of the spermatogenic wave and initial formation of the blood-testis barrier, resulting in irreversible testicular degeneration in mice. J. Reprod. Dev. *59*, 525–535.

de Rooij, D.G., and Russell, L.D. (2000). All you wanted to know about spermatogonia but were afraid to ask. J. Androl. *21*, 776–798.

del Corral, R.D., and Storey, K.G. (2004). Opposing FGF and retinoid pathways: a signaling switch that controls differentiation and patterning onset in the extending vertebrate body axis. Bioessays *26*, 857–869.

DeLeve, L.D., and Wang, X. (2000). Role of oxidative stress and glutathione in busulfan toxicity in cultured murine hepatocytes. Pharmacology *60*, 143–154.

Drumond, A.L., Weng, C.C., Wang, G., Chiarini-Garcia, H., Eras-Garcia, L., and Meistrich, M.L. (2011). Effects of multiple doses of cyclophosphamide on mouse testes: accessing the germ cells lost, and the functional damage of stem cells. Reprod. Toxicol. *32*, 395–406.

Ebata, K.T., Zhang, X., and Nagano, M.C. (2005). Expression patterns of cell-surface molecules on male germ line stem cells during postnatal mouse development. Mol. Reprod. Dev. *72*, 171–181.

Gewiss, R.L., Schleif, M.C., and Griswold, M.D. (2021). The role of retinoic acid in the commitment to meiosis. Asian J. Androl. *23*, 549–554.

Grisanti, L., Falciatori, I., Grasso, M., Dovere, L., Fera, S., Muciaccia, B., Fuso, A., Berno, V., Boitani, C., Stefanini, M., and Vicini, E. (2009). Identification of spermatogonial stem cell subsets by morphological analysis and prospective isolation. Stem Cell. *27*, 3043–3052.

Günzel, D., and Yu, A.S.L. (2013). Claudins and the modulation of tight junction permeability. Physiol. Rev. *93*, 525–569.

Haenisch, M., Nguyen, T., Fihn, C.A., Goldstein, A.S., Amory, J.K., Treuting, P., Brabb, T., and Paik, J. (2021). Investigation of an ALDH1A1-specific inhibitor for suppression of weight gain in a diet induced mouse model of obesity. Int. J. Obes. *45*, 1542–1552.

Hasegawa, K., and Saga, Y. (2012). Retinoic acid signaling in Sertoli cells regulates organization of the blood-testis barrier through cyclical changes in gene expression. Development *139*, 4347–4355.

Holczbauer, Á., Gyöngyösi, B., Lotz, G., Szijártó, A., Kupcsulik, P., Schaff, Z., and Kiss, A. (2013). Distinct claudin expression profiles of hepatocellular carcinoma and metastatic colorectal and pancreatic carcinomas. J. Histochem. Cytochem. *61*, 294–305.

Ishii, K., Ishiai, M., Morimoto, H., Kanatsu-Shinohara, M., Niwa, O., Takata, M., and Shinohara, T. (2014). The Trp53-Trp53inp1-Tnfrsf10b pathway regulates the radiation response of mouse spermatogonial stem cells. Stem Cell Rep. *3*, 676–689.

Jauregui, E.J., Mitchell, D., Topping, T., Hogarth, C.A., and Griswold, M.D (2018). Retinoic acid receptor signaling is necessary in steroidogenic cells for normal spermatogenesis and epididymal function. Development *145*, dev160465.

Kanatsu-Shinohara, M., Ogonuki, N., Inoue, K., Miki, H., Ogura, A., Toyokuni, S., and Shinohara, T. (2003). Long-term proliferation in culture and germline transmission of mouse male germline stem cells. Biol. Reprod. *69*, 612–616.

Kanatsu-Shinohara, M., Inoue, K., Miki, H., Ogonuki, N., Takehashi, M., Morimoto, T., Ogura, A., and Shinohara, T. (2006). Clonal origin of germ cell colonies after spermatogonial transplantation in mice. Biol. Reprod. *75*, 68–74.

Kanatsu-Shinohara, M., Inoue, K., Takashima, S., Takehashi, M., Ogonuki, N., Morimoto, H., Nagasawa, T., Ogura, A., and Shinohara, T. (2012). Reconstitution of mouse spermatogonial stem cell niches in culture. Cell Stem Cell *11*, 567–578.

Kanatsu-Shinohara, M., and Shinohara, T. (2013). Spermatogonial stem cell self-renewal and development. Annu. Rev. Cell Dev. Biol. *29*, 163–187.

Kanatsu-Shinohara, M., Naoki, H., and Shinohara, T. (2016). Nonrandom germline transmission of mouse spermatogonial stem cells. Dev. Cell *38*, 248–261.

Kanatsu-Shinohara, M., Ogonuki, N., Matoba, S., Ogura, A., and Shinohara, T. (2020). Autologous transplantation of spermatogonial stem cells restores fertility in congenitally infertile mice. Proc. Natl. Acad. Sci. USA *117*, 7837–7844.

Kanatsu-Shinohara, M., Shiromoto, Y., Ogonuki, N., Inoue, K., Hattori, S., Miura, K., Watanabe, N., Hasegawa, A., Mochida, K., Yamamoto, T., et al. (2023). Intracytoplasmic sperm injection induces transgenerational abnormalities in mice. J. Clin. Invest. *133*, e170140.

Kent, T., Arnold, S.L., Fasnacht, R., Rowsey, R., Mitchell, D., Hogarth, C.A., Isoherranen, N., and Griswold, M.D. (2016). ALDH enzyme expression is independent of the spermatogenic cycle, and their inhibition causes misregulation of murine spermatogenic processes. Biol. Reprod. *94*, 12–13.

Kubota, H., and Brinster, R.L. (2018). Spermatogonial stem cells. Biol. Reprod. *99*, 52–74.

McLean, D.J., Russell, L.D., and Griswold, M.D. (2002). Biological activity and enrichment of spermatogonial stem cells in vitamin A-deficient and hyperthermia-exposed testes from mice based on colonization following germ cell transplantation. Biol. Reprod. *66*, 1374–1379.

Meistrich, M.L., and van Beek, M.E.A.B. (1993). Spermatogonial stem cells. In Cell and molecular biology of the testis, C.C. Desjardins and L.L. Ewing, eds. (Oxford University Press), pp. 266–295.

Morimoto, H., Ogonuki, N., Kanatsu-Shinohara, M., Matoba, S., Ogura, A., and Shinohara, T. (2021). Spermatogonial stem cell transplantation into nonablated mouse recipient testes. Stem Cell Rep. *16*, 1832–1844.

Morimoto, H., Kanatsu-Shinohara, M., and Shinohara, T. (2023). WIN18,446 enhances spermatogonial stem cell homing and fertility after germ cell transplantation by increasing blood-testis barrier permeability. J. Reprod. Dev. *69*, 347–355.

Morrow, C.M.K., Tyagi, G., Simon, L., Carnes, K., Murphy, K.M., Cooke, P.S., Hofmann, M.-C.C., and Hess, R.A. (2009). Claudin 5 expression in mouse seminiferous epithelium is dependent upon the transcription factor Ets variant 5 and contributes to blood-testis barrier function. Biol. Reprod. *81*, 871–879.

Myers, A.L., Kawedia, J.D., Champlin, R.E., Kramer, M.A., Nieto, Y., Ghose, R., and Andersson, B.S. (2017). Clarifying busulfan metabolism and drug interactions to support new therapeutic drug monitoring strategies: a comprehensive review. Expet Opin. Drug Metabol. Toxicol. *13*, 901–923.

Nagano, M., Avarbock, M.R., and Brinster, R.L. (1999). Pattern and kinetics of mouse donor spermatogonial stem cell colonization in recipient testes. Biol. Reprod. *60*, 1429–1436.

Nakamura, Y., Jörg, D.J., Kon, Y., Simons, B.D., and Yoshida, S. (2021). Transient suppression of transplanted spermatogonial stem cell differentiation restores fertility in mice. Cell Stem Cell *28*, 1443–1456.e7.

Oatley, M.J., Racicot, K.E., and Oatley, J.M. (2011). Sertoli cells dictate spermatogonial stem cell niches in the mouse testis. Biol. Reprod. *84*, 639–645.

Ogawa, T., Aréchaga, J.M., Avarbock, M.R., and Brinster, R.L. (1997). Transplantation of testis germinal cells into mouse seminiferous tubules. Int. J. Dev. Biol. *41*, 111–122.

Ogawa, T., Dobrinski, I., and Brinster, R.L. (1999). Recipient preparation is critical for spermatogonial transplantation in the rat. Tissue Cell *31*, 461–472.

Ogawa, T., Ohmura, M., Yumura, Y., Sawada, H., and Kubota, Y. (2003). Expansion of murine spermatogonial stem cells through serial transplantation. Biol. Reprod. *68*, 316–322.

Ogura, A., Ogonuki, N., Miki, H., and Inoue, K. (2005). Microinsemination and nuclear transfer using male germ cells. Int. Rev. Cytol. *246*, 189–229.

Paik, J., Haenisch, M., Muller, C.H., Goldstein, A.S., Arnold, S., Isoherranen, N., Brabb, T., Treuting, P.M., and Amory, J.K. (2014). Inhibition of retinoic acid biosynthesis by the Bisdichloroacetyldiamine WIN 18,446 markedly suppresses spermatogenesis and alters retinoid metabolism in mice. J. Biol. Chem. *289*, 15104–15117.

Schofield, R. (1978). The relationship between the spleen colony-forming cell and the haemopoietic stem cell. Blood Cell *4*, 7–25.

Shashikanth, N., France, M.M., Xiao, R., Haest, X., Rizzo, H.E., Yeste, J., Reiner, J., and Turner, J.R. (2022). Tight junction channel regulation by interclaudin interference. Nat. Commun. *13*, 3780.

Shinohara, T., Orwig, K.E., Avarbock, M.R., and Brinster, R.L. (2001). Remodeling of the postnatal testis is accompanied by dramatic changes in stem cell number and niche accessibility. Proc. Natl. Acad. Sci. USA *98*, 6186–6191.

Takashima, S., Kanatsu-Shinohara, M., Tanaka, T., Takehashi, M., Morimoto, H., and Shinohara, T. (2011). Rac mediates mouse spermatogonial stem cell homing to germline niches by regulating transmigration through the blood-testis barrier. Cell Stem Cell *9*, 463–475.

Tikhonova, A.N., Lasry, A., Austin, R., and Aifantis, I. (2020). Cell-by-cell deconstruction of stem cell niches. Cell Stem Cell *27*, 19–34.

Tokuda, M., Kadokawa, Y., Kurahashi, H., and Marunouchi, T. (2007). CDH1 is a specific marker for undifferentiated spermatogonia in mouse testes. Biol. Reprod. *76*, 130–141.

Wu, S., Yan, M., Ge, R., and Cheng, C.Y. (2020). Crosstalk between Sertoli and germ cells in male fertility. Trends Mol. Med. *26*, 215–231.

Yoshinaga, K., Nishikawa, S., Ogawa, M., Hayashi, S., Kunisada, T., Fujimoto, T., and Nishikawa, S. (1991). Role of c-kit in mouse spermatogenesis: identification of spermatogonia as a specific site of c-kit expression and function. Development *113*, 689–699.

Stem Cell Reports, Volume *19*

Supplemental Information

Restoration of fertility in nonablated recipient mice after spermatogonial stem cell transplantation

Hiroko Morimoto, Narumi Ogonuki, Shogo Matoba, Mito Kanatsu-Shinohara, Atsuo Ogura, and Takashi Shinohara

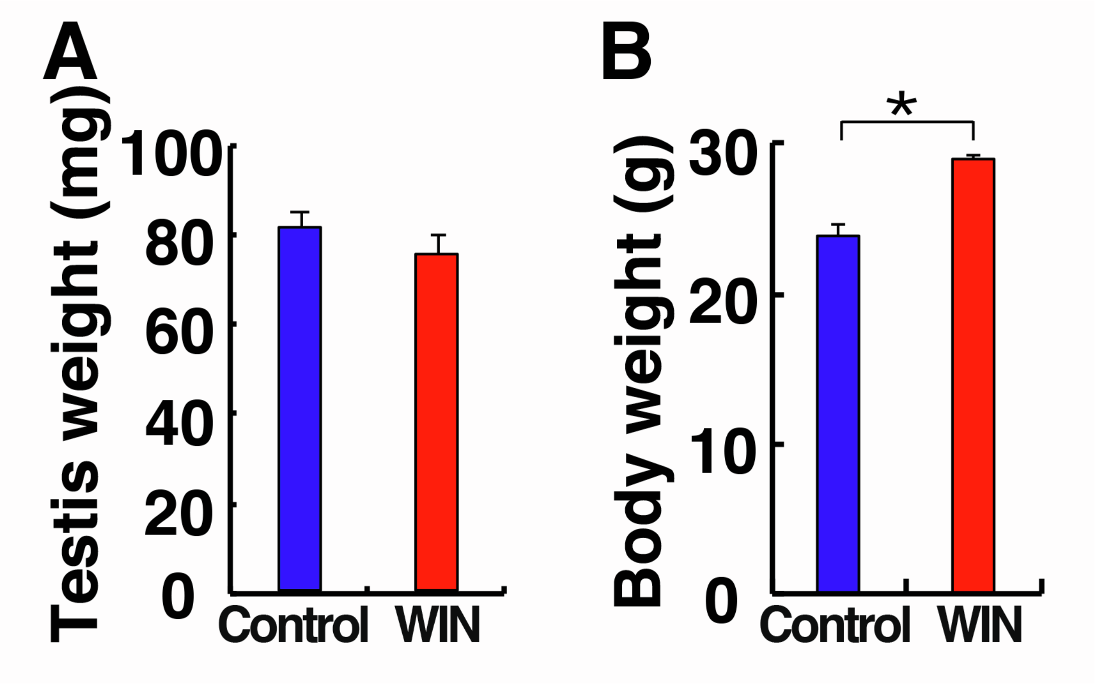

Supplemental Figure S1 The impact of WIN on testis and body size. (A) Testis weight (n = 6), (B) Body weight (n = 3).

Supplemental Table S1 Antibodies used in the present study

name	company	catalog number	application
Mouse anti ACTB monoclonal antibody	Sigma Aldrich: St. Lois, MO	A5441	Western blot (1.4 μg /ml)
Rabbit anti mouse CLDN3 polyclonal antibody	Thermo Fisher: Rockford, IL	34-1700	Immunostaining Western blot (0.5 μg /ml)
Rabbit anti human CLDN5 monoclonal antibody	abcam : Cambridge, UK	ab131259	Western blot (2.6 μg /ml)
Rabbit human CLDN5 polyclonal antibody	abcam : Cambridge, UK	ab15106	Immunostaining (1/200)
Rabbit anti human CLDN11 polyclonal antibody	abcam : Cambridge, UK	ab53041	Immunostaining Western blot (1.0 μg /ml)
Rabbit anti human CXCL12 polyclonal antibody	SantaCruz : Dallas, TX	sc-28876	Immunostaining Western blot (2.0 μg /ml)
Goat anti human FGF2 polyclonal antibody	SantaCruz : Dallas, TX	sc-1390	Immunostaining (2.0 μg /ml)
Mouse anti human FGF2 monoclonal antibody	SantaCruz : Dallas, TX	sc-74412	Western blot (0.5 μg /ml)
Rabbit anti human GDNF polyclonal antibody	SantaCruz : Dallas, TX	sc-328	Immunostaining (0.25 μg /ml)
Rabbit anti mouse GDNF polyclonal antibody	abcam : Cambridge, UK	ab18956	Western blot (5.0 μg /ml)
Rabbit anti-mouse OCLN	Gift from Dr. Sachiko Tsukita (Osaka Univ)	Not applicable	Immunostaining (1.0 μg /ml))

Mouse anti human OCLN monoclonal antibody	SantaCruz : Dallas, TX	sc-133256	Western blot (0.5 µg /ml)
Rabbit anti mouse SYCP3	NOVUS, Centennial, CO,	NB300-231	Immunostaining (10.0 µg /ml)
APC streptavidin	Biolegend : San Diego, CA	#405207	Biotin Tracer (0.2 µg /ml)
Alexa Fluor 488 donkey anti rat IgG	Thermo Fisher: Rockford, IL	A21208	Immunostaining (2.0 µg /ml)
Alexa Fluor 488 donkey anti goat IgG	Thermo Fisher: Rockford, IL	A11055	Immunostaining (2.0 µg /ml)
Alexa Fluor 555 donkey anti mouse IgG	Thermo Fisher: Rockford, IL	A31571	Immunostaining (2.0 µg /ml)
Alexa Fluor 555 donkey anti rabbit IgG	Thermo Fisher: Rockford, IL	A31573	Immunostaining (2.0 µg /ml)
HRP-anti rabbit IgG	Cell signaling: Danvers, MA	#7074	Western blot (1/2000)
HRP-anti muse IgG	Cell signaling: Danvers, MA	#7076	Western blot (1/2000)
Rhodamine conjugated Peanut agglutinin	Vector laboratories: Burlingame, CA	#RL-1072-5	Immunostaining (1/500)

Supplemental Table S2 PCR primers used in the present study

primer	forward	reverse
Cldn3	CACCACTACCAGCAGTCGATGAAC	AGACTGTGTGTCGTCTGTCACCATC
Cldn5	TAACCTGAAAGGGCAGCTGGAGAAAC	AGGGTCCAGGCTAAGTCCTTTGGTTCAGTAG
Cldn11	CTGCCGAAAAATGGACGAACTG	TGCACGTAGCCTGGAAGGATGA
Cxcl12	GCTCTGCATCAGTGACG	CCAGGTACTCTTGGATCC
Fgf2	CTCTACTGCAAGAACGGCG	CATAGCAAGGTACCGGTTGG
Gdnf	GCCACTTGGAGTTAATGTCC	CTTCGAGAAGCCTCTTACCG
Hprt	GCTGGTGAAAAGGACCTCT	CACAGGACTAGAACACCTGC
Ocln	TTGGGACAGAGGCTATGG	ACCCACTCTTCAACATTGGG

Supplemental experimental procedure

Analysis of recipient testes

Recipient mice were sacrificed 2 months after transplantation, and the number of donor cell-derived colonies was counted under UV light. Clusters of germ cells were considered as colonies when they exceeded a length of 0.1 mm and extended across the entire basement membrane of the seminiferous tubules, as previously defined (Nagano et al., 1999). For evaluation of regeneration, two histological sections were prepared from the testes and epididymides of each animal. The number of tubule cross-sections showing spermatogenesis (defined as the presence of multiple layers of germ cells in entire circumference of the seminiferous tubules) was recorded by counting all the tubules on the section. For epididymal tubule count, tubules containing any sperm were recorded. Perimeter was determined by cellSens (Olympus, Tokyo, Japan).

Tracer experiment

Biotin tracer experiments were conducted following established procedures (Takashima et al., 2011). In brief, sulfo-NHS-LC-biotin solution (7.5 mg/ml; Thermo Fisher Scientific, Waltham, MA) was prepared in phosphate-buffered saline (PBS) containing 1 mM $CaCl_2$. Approximately 10 μl of the biotin solution was microinjected into the interstitium of adult (> 8-week-old) testes using a syringe with 30 G disposable needles. After 30 min, the testes were collected and fixed in 4% paraformaldehyde. The samples were frozen in liquid nitrogen for cryosectioning. Frozen sections were prepared

for further staining by allophycocyanin (APC)-conjugated streptavidin (Biolegend, San Diego, CA). Endogenous biotin staining was examined by incubation with APC-conjugated streptavidin without biotin injection.

Immunostaining

Testis samples were fixed in 4% paraformaldehyde for 2 h at 4°C, and embedded in Tissue-Tek OCT compound (Sakura Finetek, Tokyo, Japan) for cryosectioning. Sections of 8 μm thickness were made. To block non-specific antibodies, sections were treated with 3% bovine serum albumin (BSA) and 10% goat serum in PBS supplemented with 0.1% Tween 20 (PBST) for 1 h at room temperature. The sections were then incubated with indicated primary and secondary antibodies with 0.5 % BSA in PBST, overnight and for 1 h, respectively. Thirty tubules were counted from at least two mice. Rhodamine-labeled PNA (Vector Laboratories, Burlingame, CA) was used to detect the acrosome. Antibodies used are listed in Table S1. Hoechst 33342 (Sigma) was used for counterstaining. Staining without the primary antibodies were used as negative controls.

Real-time PCR analysis

Total RNA was isolated using TRIzol reagent (Invitrogen, Carlsbad, CA). First-strand cDNA was produced using a Verso cDNA synthesis kit for reverse transcription-PCR (Thermo Fisher Scientific). For real-time PCR, CFX connect™ real-time PCR system (Bio-Rad, Hercules, CA) and *Power* SYBR Green PCR Master Mix (Applied Biosystems) were utilized according to the manufacturers' protocols. Transcript levels

were normalized to those of *Hprt*. The PCR conditions were as follows: 95°C for 10 min, followed by 40 cycles at 95°C for 15 s and 60°C for 1 min. Each PCR was performed at least in triplicate. Primers used are listed in Table S2.

Western blotting

Samples were separated by SDS-PAGE, transferred to Immobilon-P membranes (Millipore, Burlington, MA), and incubated with primary antibodies. The antibodies used in the experiments are shown in the Table S1.

Microinsemination

EGFP-expressing seminiferous tubules were collected, and the germ cells were mechanically extracted from the tubules using fine forceps. Microinsemination was performed as described previously using elongated spermatids and sperm (Ogonuki et al., 2006). Embryos that reached the two-cell stage after 24 h in culture were transferred to the oviducts of day-1 pseudopregnant ICR female mice. Fetuses were retrieved on day 19.5 by caesarean section.

Supplemental references

Nagano, M., Avarbock, M. R., and Brinster, R. L. (1999). Pattern and kinetics of mouse donor spermatogonial stem cell colonization in recipient testes. Biol. Reprod. *60*, 1429-1436.

Ogonuki, N., Mochida, K., Miki, H., Inoue, K., Fray, M., Iwaki, T., Moriwaki, K., Obata, Y., Morozumi, K., Yanagimachi, R., and Ogura, A. (2006). Spermatozoa and spermatids retrived from frozen reproductive organs or frozen whole bodies of male mice can produce offspring. Proc. Natl. Acad. Sci. USA *103*, 13098-13103.

Takashima, S., Kanatsu-Shinohara, M., Tanaka, T., Takehashi, M., Morimoto, H., and Shinohara, T. (2011). Rac mediates mouse spermatogonial stem cell homing to germline niches by regulating transmigration through the blood-testis barrier. Cell Stem Cell *9*, 463-475.
